# Co(II)-Chelated Polyimines as Oxygen Reduction Reaction Catalysts in Anion Exchange Membrane Fuel Cells

**DOI:** 10.3390/membranes13090769

**Published:** 2023-08-30

**Authors:** Yu-Chang Huang, Yen-Zen Wang, Tar-Hwa Hsieh, Ko-Shan Ho

**Affiliations:** 1 Department of Chemical and Materials Engineering, National Kaohsiung University of Science and Technology, 415, Chien-Kuo Road, Kaohsiung 80782, Taiwan; 2 Department of Chemical and Materials Engineering, National Yu-Lin University of Science & Technology, 123, Sec. 3, University Road, Dou-Liu City, Yun-Lin 64301, Taiwan

**Keywords:** ORR catalyst, Co-chelated, polynaphthalene imine, cathode catalyst, AEMFC

## Abstract

In this paper, a cobalt (Co)-chelated polynaphthalene imine (Co-PNIM) was calcined to become an oxygen reduction reaction (ORR) electrocatalyst (Co-N-C) as the cathode catalyst (CC) of an anion exchange membrane fuel cell (AEMFC). The X-ray diffraction pattern of CoNC-1000A900 illustrated that the carbon matrix develops clear C(002) and Co(111) planes after calcination, which was confirmed using high-resolution TEM pictures. Co-N-Cs also demonstrated a significant ORR peak at 0.8 V in a C–V (current vs. voltage) curve and produced an extremely limited reduction current density (5.46 mA cm^−2^) comparable to commercial Pt/C catalysts (5.26 mA cm^−2^). The measured halfway potential of Co-N-C (0.82 V) was even higher than that of Pt/C (0.81 V). The maximum power density (P_max_) of the AEM single cell upon applying Co-N-C as the CC was 243 mW cm^−2^, only slightly lower than that of Pt/C (280 mW cm^−2^). The Tafel slope of CoNC-1000A900 (33.3 mV dec^−1^) was lower than that of Pt/C (43.3 mV dec^−1^). The limited reduction current density only decayed by 7.9% for CoNC-1000A900, compared to 22.7% for Pt/C, after 10,000 redox cycles.

## 1. Introduction

The oxygen reduction reaction (ORR), which occurs in a cathode, is the bottleneck reaction of a hydrogen fuel cell, producing power via an electrochemical redox reaction. In other words, once we are able to enhance the ORR in the cathode with suitable catalysts, the power productivity of the fuel cell will be improved. Elements, like Pt [1,2,3,4,5], or metal oxides, like CoO [6,7,8], are often used as catalysts that can improve fuel cells’ ORR and power density. These Pt electrocatalysts must implant on or be in contact with the carbonaceous conducting medium to catalyze the ORR when O_2_ is supplied to the cathode. No chemical bonding on the carbon matrix surface can firmly catch the Pt catalyst particles. These Pt catalyst particles can easily detach from the matrix during the purification or activation of the catalysts. For example, CoO can be dissolved and become Co(II) ions when strong acids, like sulfuric acid, are used to wash the catalysts.

To solve these problems with the particle catalysts, single-atom catalysts (SACs), in which catalytic transition metals are covalently bonded in the carbonaceous network, were prepared through high-temperature calcination [9,10,11,12,13,14,15].

Several macrocyclic compounds with nitrogen-containing functional groups, like pyrrole and pyridine, were used to capture metal ions, like Co(II), inside the ring. In addition, the so-called metal–organic frameworks (MOFs) were constructed. However, these metal-complexed MOFs typically degrade into small pieces and evaporate during the process of calcination. This means that we must mix them with other carbon media, like carbon black (CB), before calcination to protect them from degradation and simultaneously develop the necessary conducting matrix [16,17,18,19]. Above all, CoS_2_-N-doped reduced graphene oxide decorated with Pt nanodendrite was applied as an effective anode in the electro-oxidation of various alcohols with different numbers of carbons [20].

We published a series of aromatic nitrogen-containing (N-containing) polymers (ANCPs), like polyanilines (PANI) [3] and aromatic polyamines (APIM) [21,22], that can be used to capture Co(II) before calcination. These Co(II)-chelated ANCPs can facilely convert into Co, N-doped carbonaceous catalysts (Co-N-Cs) via calcination alone, without adding CB or preparing a MOF system before calcination.

In order to chelate more Co(II), we prepared an APIM with 1,8 diamino naphthalene (DAN) with side-amino groups and more aromatic rings. These side-amino groups can catch more Co(II), and the use of naphthalene guarantees an effectively conducting carbon substrate in the prepared Co-N-Cs.

## 2. Materials and Methods

### 2.1. Materials

The materials used in this study were DAN (1,8 diaminonaphthalene) (Tokyo Kasei Kogyo Co., Ltd., Tokyo, Japan), TPAL (terephthalaldehyde) (Tokyo Kasei Kogyo Co., Ltd., Tokyo, Japan), anhydrous cobalt(II) chloride (CoCl_2_, J.T. Baker, Radnor, PA, USA), and DGBE (diethylene glycol monobutyl ether) (Eastman Chem. Co., Ltd., Kingsport, TN, USA).

### 2.2. Preparation of Polynaphthalene Imine (PNIM)

A total of 2.37 g of 1,8-diaminonaphthalene (DAN) was mixed in 20 g of diethylene glycol monobutyl ether (DGBE), followed by stirring to dissolve it at room temperature. A total of 1.34 g of terephthalaldehyde (TPAL) was dissolved in 20 g of DGBE via stirring at 80 °C. We dissolved 0.65 g of anhydrous cobalt chloride (CoCl_2_) in 10 g of deionized water. The DAN solution was added to the TPAL solution and stirred to uniform. The CoCl_2_ solution was introduced into the solution, and stirring continued. The polymerization reaction continued evenly at 80 °C for 24 h, followed by centrifugation at 2500 rpm for 5 min. After separating the precipitate, we placed it in a glass Petri dish and dried the solvent at a constant temperature of 80 °C in a vacuum oven for 24 h. After drying, the catalyst precursor Co-complexed polyimines were obtained and named Co-PNIM.

In a high-purity nitrogen environment, the precursor Co-PNIM was calcined at 800 °C (or at 900 °C or 1000 °C). The temperature was increased from RT to the target temperature at 10 °C min^−1^ and maintained for 30 min. As the obtained calcined powders contained apparent magnetism, separating the magnetized powders via grinding was impossible. For continued characterization, acid washing with 1 M sulfuric acid solution was required to remove the magnetic materials and weaken the magnetism. Acid washing was performed at 60 °C for 24 h, followed by washing with deionized water, and the powder was dried in an oven at 80 °C for 8 h. Then, we performed the second calcination at 700 °C (or at 800 °C or 900 °C) under a mixed gas environment of nitrogen and ammonia. We set the second calcination temperature as 100 °C lower than the first calcination temperature, and the calcination time remained the same as the first time. After the sample temperature dropped to RT, the catalyst was obtained and ready for characterization.

### 2.3. Characterization

#### 2.3.1. Fourier Transform Infrared Spectroscopy (FTIR)

A total of 5 mg samples were mixed with KBr powders (dried in an oven) and ground into powders, which were pressed into a tablet for IR measurement. The spectrum ranged from 4000 cm^−1^ to 400 cm^−1^, with a total of 16 scannings having been performed. The measuring resolution was set at 4 cm^−1^.

#### 2.3.2. X-ray Diffraction Spectroscopy (XRD)

A copper target (Cu-Kα) Rigaku X-ray source (Rigaku, Tokyo, Japan), which produces X-rays with a wavelength of 1.5402 Å, was used to construct the diffraction patterns of the Co-N-Cs. The scanning angle (2θ) ranged from 10° to 90° and was set at 40 kV (voltage) and 30 mA (current). The scanning rate was 1° min^−1^.

#### 2.3.3. Raman Spectroscopy

The Raman spectra of various Co-N-Cs were obtained though conducting Raman spectroscopy (TRIAX 320, HOBRIA, Kyoto, Japan).

#### 2.3.4. Transmission Electron Microscopy (TEM)

Micropictures of Co-N-Cs were taken using an HR-AEM field emission transmission electron microscope (HITACHI FE-2000, Hitachi, Tokyo, Japan). Samples were first added to acetone and stirred until uniform. Successively, samples were added dropwise onto copper grids coated with carbon before exposing them to electron radiation.

#### 2.3.5. BET Measurement

We obtained the nitrogen adsorption–desorption isotherms via an Autosorb IQ gas sorption analyzer (Micromeritics-ASAP2020, Norcross, GA, USA) at 25 °C. All Co-N-Cs were first vacuumed in an oven for 24 h at 100 °C. The BET surface area was obtained via the BET equation when a linear BET plot with a positive C value was found in the relative pressure range. The size distribution of pores was constructed via the quenched solid density functional theory (QSDFT), following a slit/cylinder pores model. The whole pore volumes were obtained at P/P_0_ = 0.95.

#### 2.3.6. Current–Voltage Curve (C–V) and Linear Scan Voltammetry (LSV)

First, a total of 2.9 mg of catalyst was added to a mixed solvent composed of an equal amount (357 μL) of ethanol and deionized water. Following this, 7.14 μL of Nafion proton solution was introduced. The mixture was put in an ultrasonic oscillator to homogenize become a uniform slurry. Next, 5 μL of the slurry was spread onto the glassy carbon electrode, which was installed to the rotating part of the potentiostat. Ag/AgCl and Pt wires were used as the reference and counter electrodes, respectively. Eventually, 0.1 M KOH_(aq)_ was added as the alkaline electrolyte. The oxygen was then fed into the solution for 30 min, after which the C–V and LSV curves were measured.

We constructed the Tafel curves (Tafel slope) via the data obtained from the LSV curves.

#### 2.3.7. Membrane Electrode Assembly (MEA)

First, the X37-50RT anion exchange membrane (Dioxide Materials, Raton, FL, USA) (2 cm × 2 cm) was soaked in 1 M KOH_(aq)_ at RT for 24 h. When the membrane was separated from the plastic film, the activation procedure was completed and the membrane is ready for the MEA fabrication procedure.

A total of 36 mg of the prepared Co-N-C catalyst was mixed with 800 mg of pure water in an ultrasonic oscillator and shaken for 30 min. Then, 180 mg of ionomer solution and 800 mg of methanol were added and shaken again until a uniform cathode ink was formed.

The anode slurry was prepared by mixing 18 mg of the Pt/C catalyst with 400 mg of pure water, following which it was shaken for 30 min in the ultrasonic oscillator. Following this, 900 mg of ionomer solution and 400 mg of methanol were introduced into the oscillator and shaken again for 30 min to form an anode ink. The cathode and anode inks were coated on both sides of the membrane and pressed into an MEA. The prepared MEA was then assembled into a single-cell device to measure the polarization curve and power density.

#### 2.3.8. Single-Cell Test

The MEA was then assembled on the testing station to measure the single cell’s exporting potentials and power densities under increasing current densities through a fuel cell testing instrument (model FCED-P50; Asia Pacific Fuel Cell Technologies, Ltd., Miaoli, Taiwan). The underperformance area was 2 cm × 2 cm. Temperatures of the testing parts, including the anode, cell, cathode, and humidifying gas, were set at 60 °C. The H_2_ (anode) and O_2_ (cathode) flow rates were maintained at 30 mL·min^−1^ and 60 mL·min^−1^, respectively, following the stoichiometry procedure.

## 3. Results and Discussion

### 3.1. FTIR Spectroscopy

**Figure 1:**
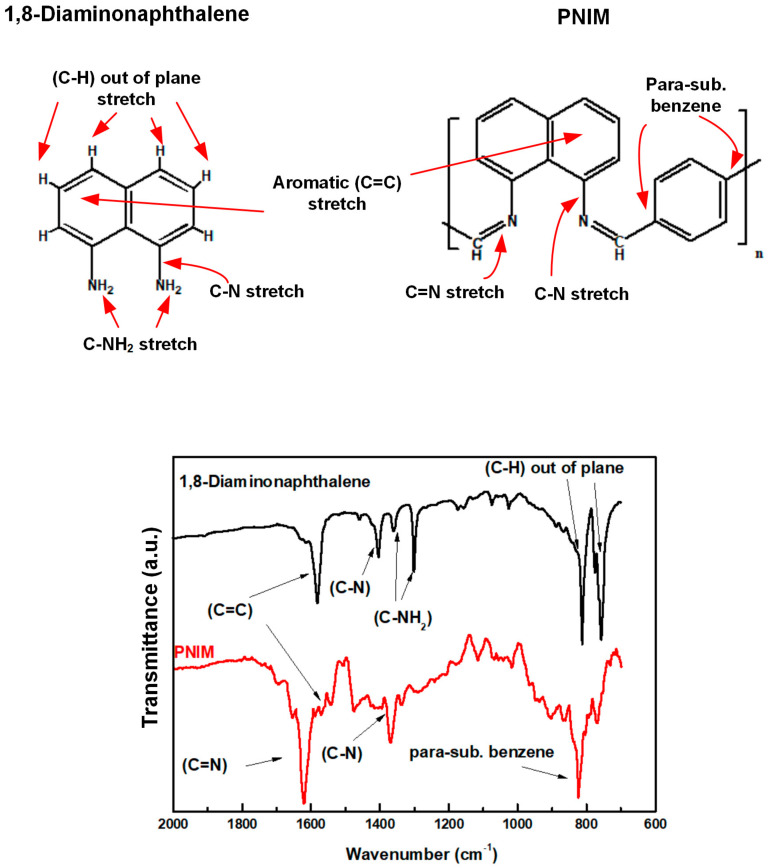
Chemical structures and IR spectra of DAN and PNIM.

The respective chemical structures and FTIR spectra of the DAN monomers and PNIM polymers are illustrated in Figure 1. Referring to the descriptive functional groups of the DANs and PNIMs in Figure 1, we can assign the corresponding peaks of each group on their IR spectra. These IR spectra also suggested that the PNIMs were successfully prepared (polymerized). The -C-N- stretch modes (at 1359 cm^−1^ and 1300 cm^−1^) [23] of the primary amines of the DAN monomer disappeared after polymerization, converting into the imine groups (-C=N-, 1619 cm^−1^) of the polymers (PNIMs). In other words, the DAN monomers successfully underwent condensation with TPAL to become PNIMs via the Schiff reaction. Moreover, a peak was observed at 830 cm^−1^, assigned as a para-substituted benzene ring of TPAL. The para-substituted benzene ring in the FTIR spectrum of PNIM indicates that TPAl joins in the polymer backbone.

### 3.2. XRD Pattern

**Scheme 1:**
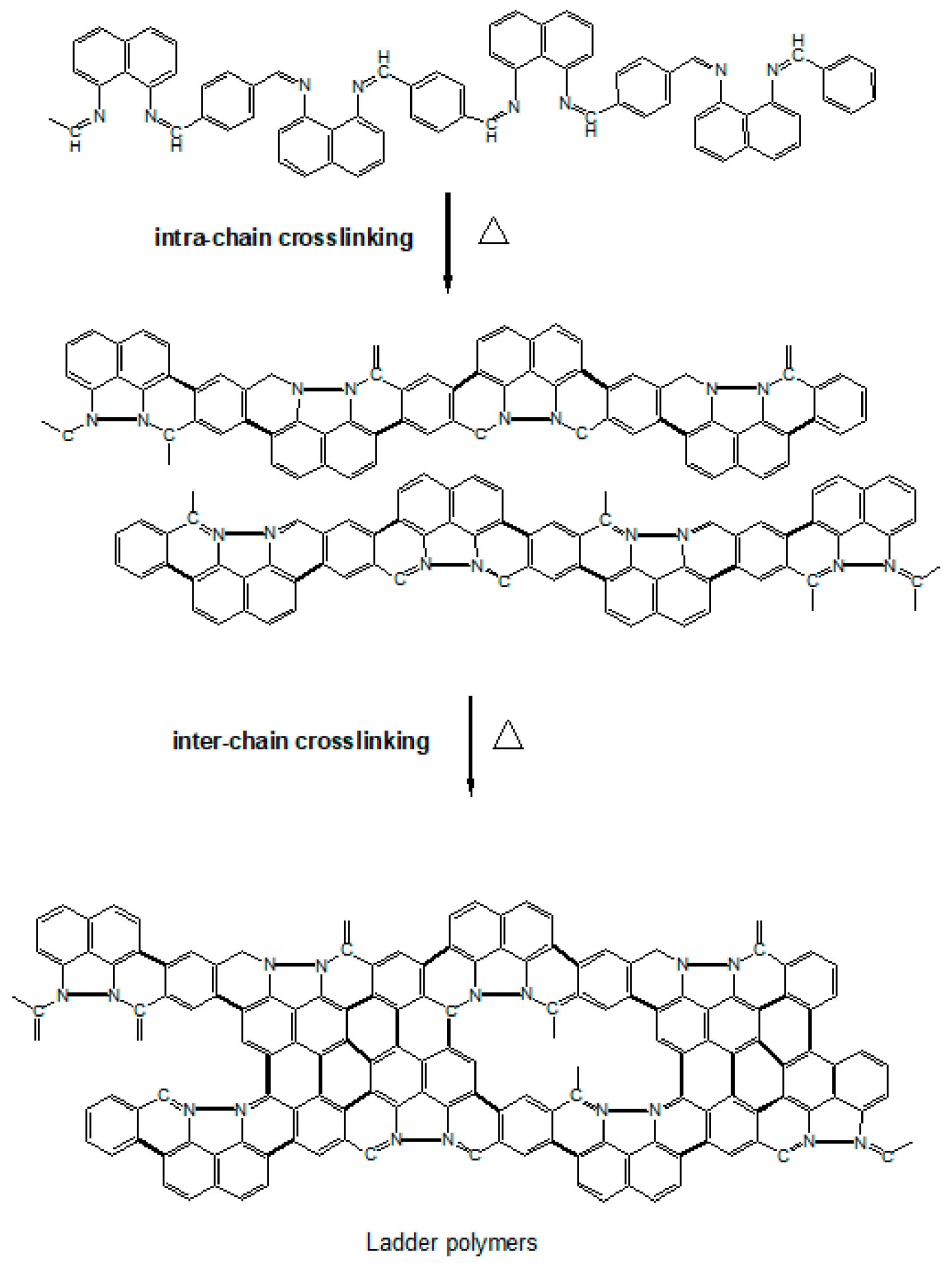
Schematic diagram of the PNIM calcination mechanism.

Referring to the chemical structure illustrated in Scheme 1, PNIM can either undergo intramolecular or intermolecular crosslinking upon thermal heating, resulting in ladder-like polymers in the initial calcination stage.

**Figure 2:**
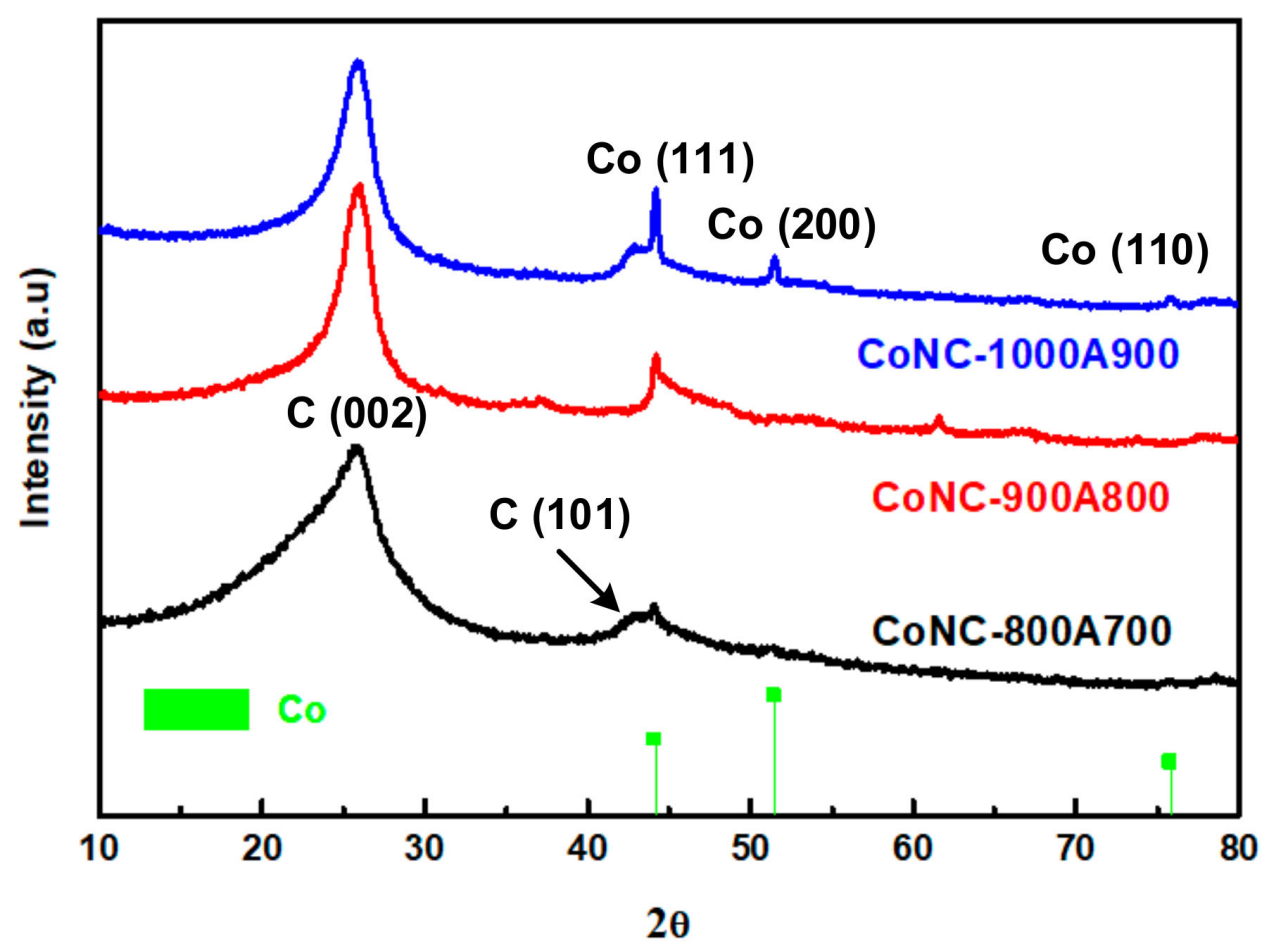
X-ray diffraction patterns of various Co-N-Cs.

These ladder polymers can then progress into a planar carbon matrix, like graphene (GF), or a tubular carbon matrix, like the carbon nanotube (CNT), in the later stage, and eventually become Co-N-Cs, as described in Scheme 1. The construction of the ordered crystalline structure of Co-N-Cs can be monitored using the X-ray patterns demonstrated in Figure 2. These intra- and inter-crosslinking reactions form the crystalline planes (C(002)) of GF or the CNT, which can be confirmed in the TEM micropictures. The C(002) peak gradually became sharper with temperatures ranging from 800 °C to 1000 °C, and the Co crystalline (Co(111)) also grew during calcination, as shown in Figure 2. Co crystallites can act as the seeds to grow a CNT, increasing the Co-N-Cs’ roughness, as found in the TEM section. With more CNTs (CNRs or CNBs) created at higher calcination temperatures, the crystallinity contributed from the carbon matrix also improved, resulting in the sharper diffraction peak of the C(002) plane. In other words, more H atoms were removed from the carbon matrix, and saturated C-C single bonds were converted into unsaturated C=C bonds during the development of the CNTs. The following discussion regarding the Raman spectra of the Co-N-Cs can illustrate the conversion of the saturated bonds into their corresponding unsaturated bonds. The XRD patterns of all types of the Co-N-Cs did not reveal the presence of any CoO crystalline peak, indicating that it was removed by acid leaching during the first calculation stage. It can be found that the attractive magnetic force of the catalyst was weakened after acid washing due to the removal of the highly magnetic CoO. Although CoO also contributes to the catalysis of the ORR, its solid magnetic attraction makes the preparation of the catalyst ink very difficult as we need to disperse the catalyst particles in the cathode ink. The formation of Co crystals during calcination, as shown in Figure 2, indicates that Co crystals can behave as seeding centers to develop the CNT structure [24,25,26,27,28]. When calcination was conducted at higher temperatures, many curved nano-rings were observed in the TEM micropictures.

### 3.3. Raman Spectroscopy

**Figure 3:**
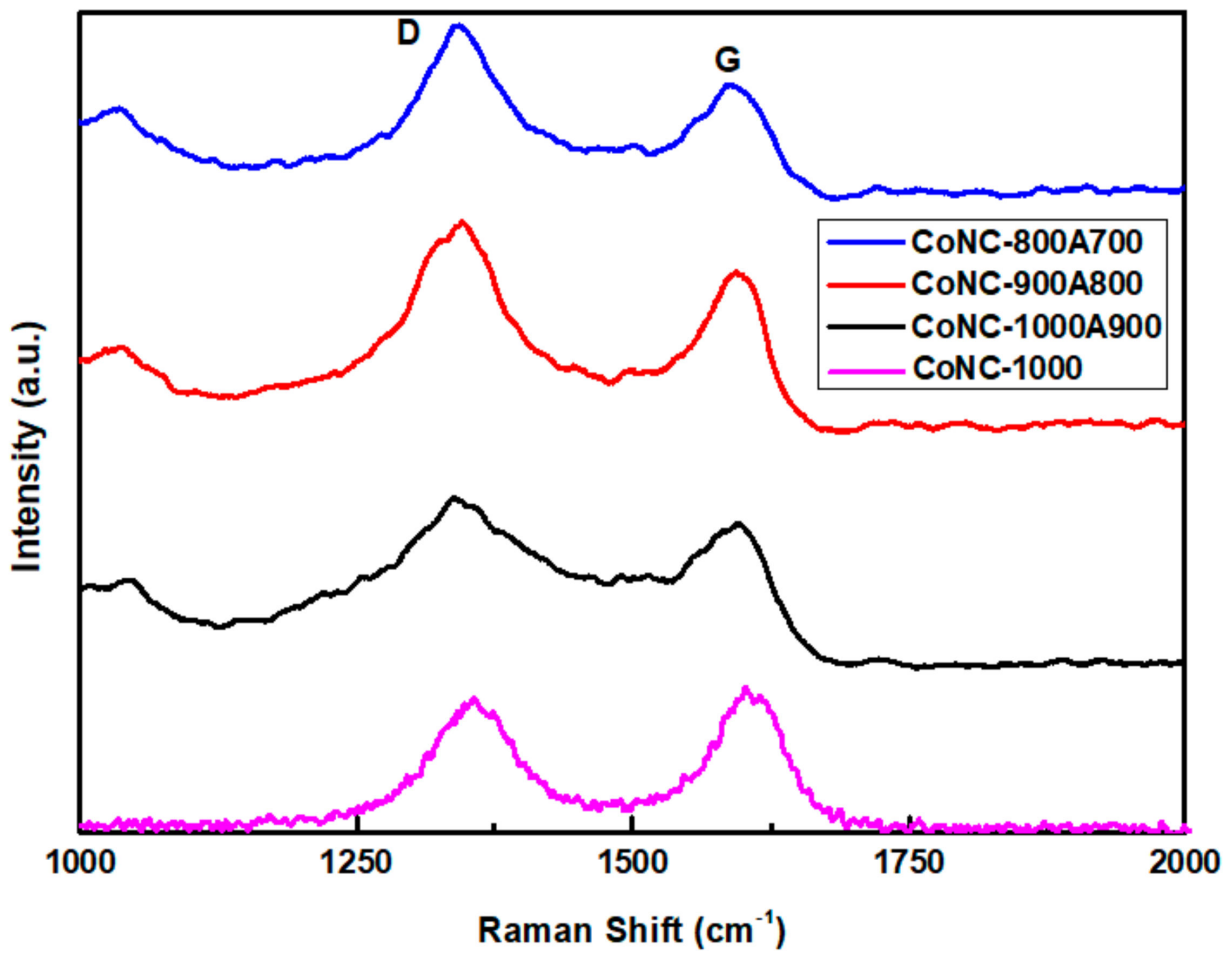
Raman spectra of various Co-N-Cs.

Scheme 1 and Figure 2 show that PNIM can be converted into a conjugated, aromatic carbon matrix through calcination. These conjugated carbons were derived from forming unsaturated C=C double bonds by consuming saturated C-C single bonds, and eventually evolved into a GF-like carbon matrix. In the Raman spectra, the absorption peaks at 1350 cm^−1^ (D band) and 1580 cm^−1^ (G band) were deemed to have been contributed from C(sp^3^) and C(sp^2^), respectively. Consequently, we were able to reveal the transformation by the variation in the respective peak intensity ratios of the D band and the G band (I_D_/I_G_) obtained from the Raman spectra in Figure 3. We realized from the X-ray diffraction patterns that more crystallized carbon structures were constructed from the more sharpened C(002) peak at higher calcination temperatures, contributing to the G-band. The more ordered structure also brings a lower resistivity (higher conductivity) of the Co-N-Cs, as listed in Table 1. When Co-PNIM only experienced the first calcination stage at 1000 °C (CoNC-1000), it contributed to the lowest I_D_/I_G_ value of 0.866, a resistivity measurement of 9.3 Ω cm (Table 1), and a high level of magnetism. This indicates that a highly crystallized structure was created at 1000 °C for CoNC-1000. The Co elements and CoO that were formed under high calcination temperatures resulted in high levels of conductivity and magnetism, which can be reduced through acid leaching, leading to a higher level of resistivity (Table 1) and less magnetism.

The significant difference observed in the Raman spectra of various Co-N-Cs was deemed to have formed from the fact that the wavenumbers of the D and G bands are not exactly the same. The shift of the G band is relevant to the thickness of the created GF, which indicates different numbers of GF layers. For the Co-N-Cs, different types (including GF, CNT, CNR, or CNB) of the crystallized carbon structures formed during calcination can contribute to the position variation of the D and G bands.

### 3.4. TEM

**Figure 4:**
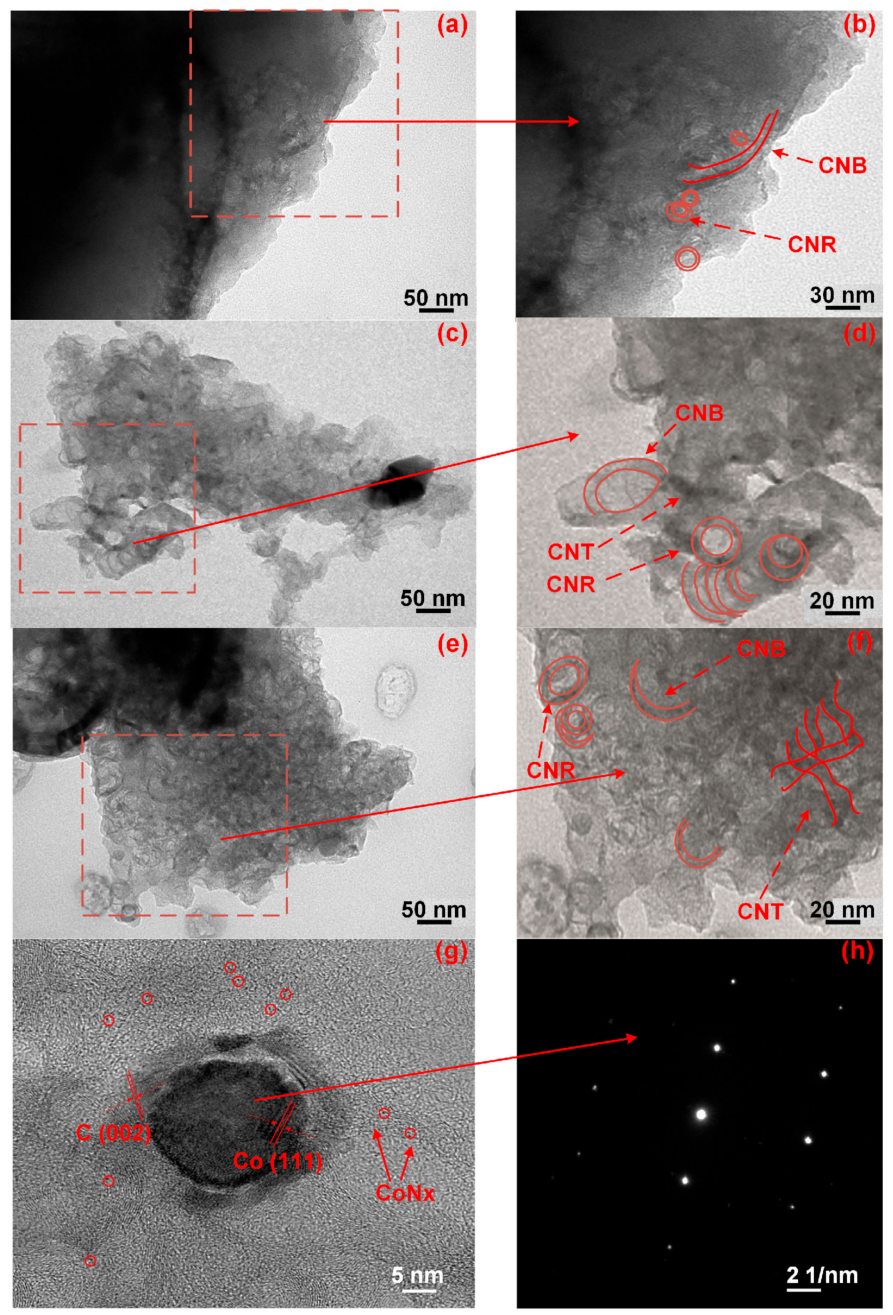
TEM micropictures of (**a**,**b**) CoNC-800A700; (**c**,**d**) CoNC-900A800; and (**e**,**f**) CoNC-1000A900. (**g**,**h**) HR-TEM and electron diffraction of a single cobalt particle of CoNC-1000A900.

The morphology of Co-N-C varied significantly when the calcination temperature was over 800 °C. In Figure 4, the carbon nano-belts (CNBs) developed under thermal treatment (>800 °C, comparing Figure 4a–c and Figure 4b–d) and their numbers increased with the calcination temperature, resulting in the formation of a rougher surface and a higher surface area. The CNBs or CNTs (Figure 4c–f), whose formations of which have been attributed to the Co-element, behave as the substrate (seed) to grow carbon shells. They can stack on each other and become the embryonic form of the CNBs or CNTs [29,30,31]. The formation of the CNBs and CNRs can also increase the cathode catalysts’ electron conductivity, as already listed in Table 1.

The Co element can also catalyze the formation of CNBs by consuming a flatter carbon matrix (which could be GF) under a high calcination temperature. For the Co catalysts that are beneath the carbon matrix, they can also catalyze the formation of the CNBs. However, these CNBs can only emerge to the surface if the carbon matrix is not sufficiently robust. When the calcination temperature was high enough to destroy the carbon covering, the growing CNBs were able to break the covering and appear on the surfaces, resulting in the formation of more CNBs.

The HR-TEM picture of the single cobalt particle buried in the CoNC-1000A900 matrix is shown in Figure 4g. The HR-TEM micrograph in Figure 4g illustrates the presence of the Co(111) and C(002) planes, and the Co^0^ electron diffraction pattern is also displayed in Figure 4h. The tiny spots distributed in the crystallized carbon matrix around the single Co particle are the Co crystals that are protected by the carbon matrix, as shown in Figure 4g, which also contributed to the high ORR catalytic properties of CoNC-1000A900.

### 3.5. ED Mappings

**Figure 5:**
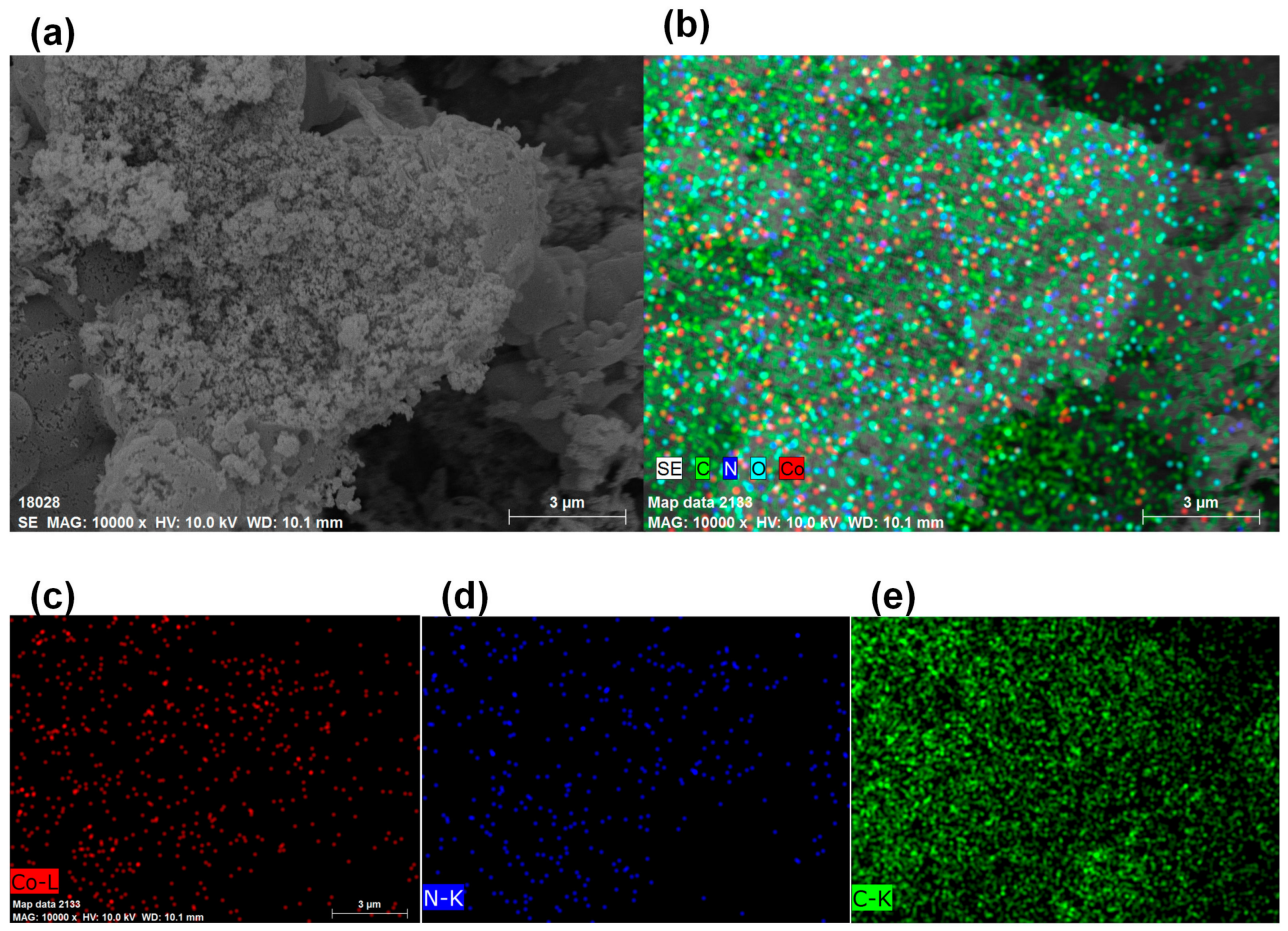
(**a**) SEM picture. (**b**) Combined Co, N, and C mappings obtained through the use of ED. (**c**) Co, (**d**) N, and (**e**) C mappings of CoNC-1000A900.

The SEM picture and the Co, N, and C element mappings of CoNC-1000A900 are illustrated in Figure 5. Figure 5a displays the highly rough surface of CoNC-1000A900, with the Co, N, and C elements dispersing homogeneously inside the catalyst. Figure 5b was separated into Figure 5c–e, representing the Co, N, and C mappings, respectively. The Co elements, including Co^0^, CoO, and CoNx, were uniformly distributed in the CoNC-1000A900 matrix, implying that many Co catalytic centers were uniformly created in the catalyst following calcination (Figure 4g and Figure 5c). In addition, the distribution pattern of N elements (Figure 5d) is similar to that of Co elements (Figure 5c), indicating that these N elements stayed close with the Co^0^, CoO, and CoNx catalytic centers. These active centers were embedded in the affluent carbon matrix (Figure 5e) that can convey electrons to the cathode to the active centers where the ORR occurs.

### 3.6. BET Surface Area and Pore Size

**Figure 6:**
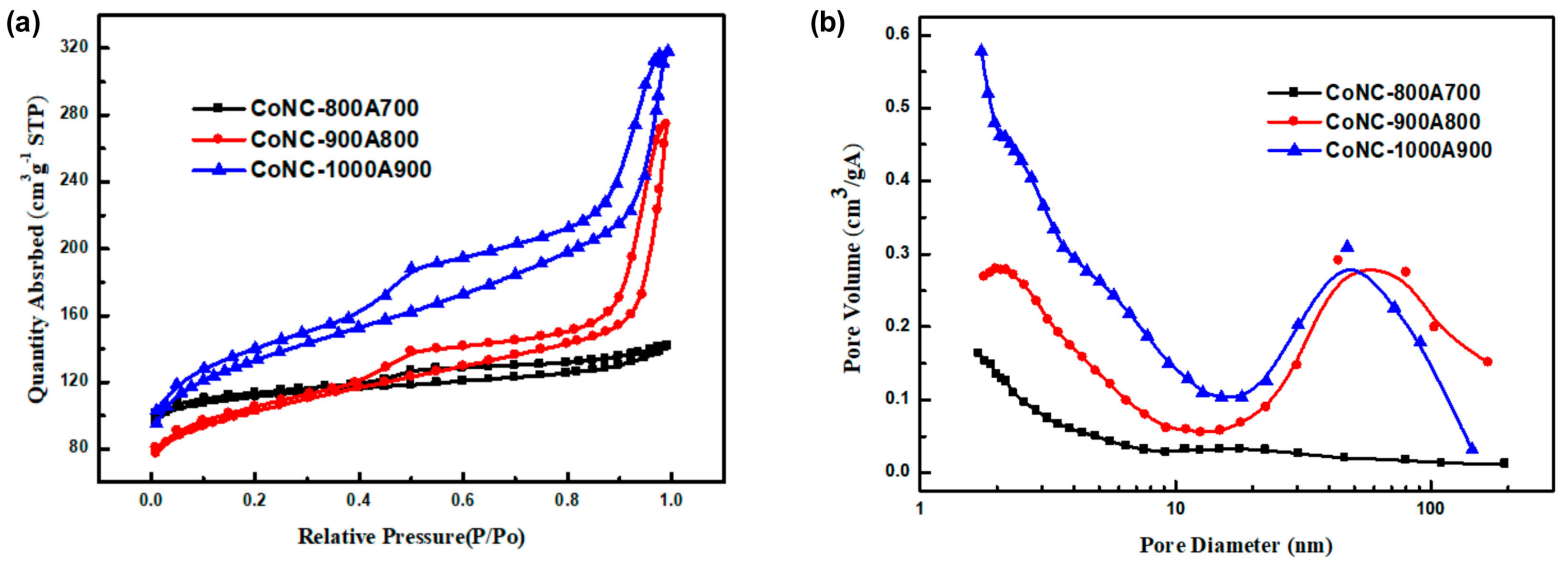
(**a**) Adsorption–desorption isotherms in N_2_ and (**b**) pore size distribution of the Co-N-Cs calcined at different temperatures.

The isothermal curves of Co-N-Cs were deemed to be similar, as according to Figure 6a. According to the IUPAC adsorption isotherm curve classification [32], this curve belongs to Type IV, which occurs when the adsorption force between the gas molecules and solids is much smaller than that of the gas molecules alone. When the gas molecules are adsorbed, the force between the adsorbed molecules will promote the adsorption capacity of other molecules, meaning that the higher the relative pressure value, the more pronounced the multi-layer adsorption phenomenon. The pore diameter distribution of the Co-N-Cs are displayed in Figure 6b. The surface area and average pore size of various Co-N-Cs are listed in Table 2.

The specific surface area (SSA) of the Co-N-Cs increased from 342.07 m^2^ g^−1^ to 437.80 m^2^ g^−1^ (Table 2) with increasing calcination temperature, resulting from the formation of rougher CNBs and CNRs, as discussed in the TEM section. The averaged pore sizes of the catalysts calcined at different temperatures were 4.09 nm, 7.4 nm, and 5.3 nm, respectively (Table 2). Figure 6b reveals that mesopores dominated in CoNC-900A800 and CoNC-100A900. The mesoporous structure is helpful for the transfer of gas molecules. CoNC-800A700 was found to have the smallest pore size among the three catalysts that were prepared under different temperatures.

### 3.7. Electrochemical Testing

#### 3.7.1. C–V and LSV Curves

**Figure 7:**
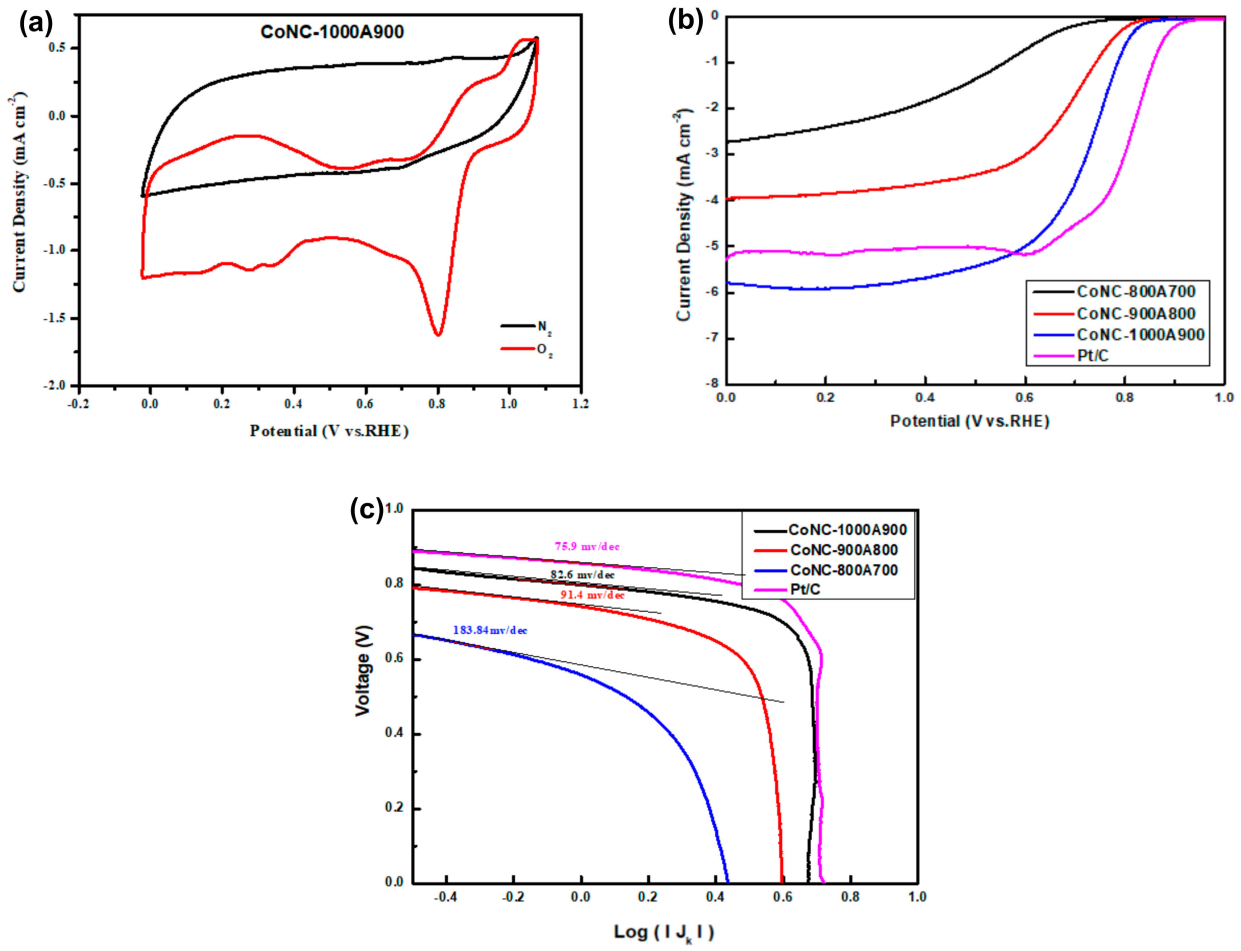
(**a**) C–V curve of CoNC-1000A900 carried out under an O_2_ and N_2_ atmosphere. (**b**) LSV curves at 1600 rpm in 0.1M KOH_(aq)_. (**c**) Tafel slopes of various Co-N-Cs.

In the C–V curve, a significant ORR peak was found at 0.8 V, as shown in Figure 7a. The presence of this reduction peak demonstrates the specific ORR capability of CoNC-1000A900 in the O_2_ atmosphere. No reduction reaction was found in the N_2_ ambient gas, indicating that the input fuel to the cathode can be replaced with air that is mainly composed of O_2_ and N_2_, and N_2_ in the air will not cause any side reduction reaction in the cathode. The active Co centers of CoNx, Co^0^, and CoO can effectively absorb the O_2_ supplied to the cathode and reduce them into hydroxyl ions (OH^−^) in the alkaline electrolyte after receiving the electrons via the external circuit. However, the ORR can be carried out in two routes, consuming different electron numbers. If the 4e-route dominates the ORR, OH^–^ is the only product. The 2e-route only consumes two electrons during the ORR, which means that the ORR is only half performed, and less power can be produced by the cell. Products of the 2e-route ORR include hydrogen peroxide (H_2_O_2_) except OH^−^.

The limited reduction current density (LRCD) obtained from the LSV curves (Figure 7b) was performed with RDE, representing the maximum reduction current that the Co-N-C cathode catalyst can produce. The LRCD of CoNC-1000A900 (5.80 mA cm^−2^), listed in Table 3, is higher than that of Pt/C (5.26 mA cm^−2^), indicating that more current could flow into the cathode, and that more power can be produced. Additionally, the half-wave potential (HWP), Tafel slope, and E_onset_ of CoNC-1000A900 are all very close to that of Pt/C, as shown in Table 3. The Tafel slopes representing the inherent reduction resistance (voltage/current) of the catalysts were obtained from the tangent lines of each curve of Figure 7c and are listed in Table 3.

In brief, the CoNC-100A900 obtained from the calcination of the Co-complexed PNIM demonstrates a significant ORR capability, a high LRCD, and a low reduction resistance (low Tafel slope) compared with Pt/C as a CC in the alkaline KOH_(aq)_ solution.

**Figure 8:**
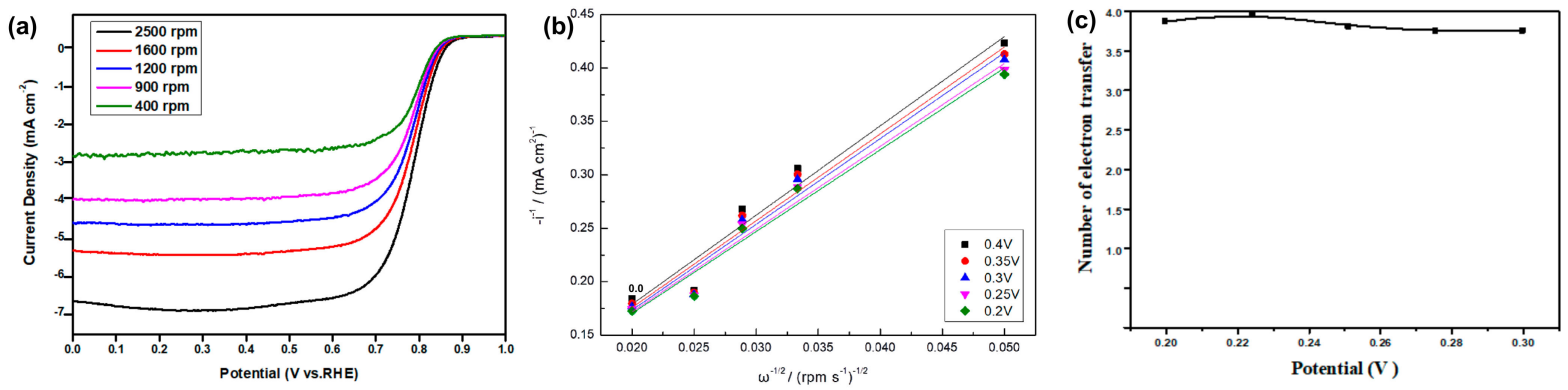
(**a**) LSV curves of CoNC-1000A900 at different rpms. (**b**) K–L lines obtained from (**a**). (**c**) Average electron-transferred number contributed from the ORR.

The electron-transferred numbers of CoNC-1000A900 during the ORR were obtained from the Koutecký–Levich (K–L) Equation (1). Based on the LSV curves (Figure 8a), we obtained K–L linear lines (Figure 8b) measured with various rpms of the RDE. K–L linear lines were then used to calculate the electron-transferred numbers under different potentials (Figure 8c) using the following equation:

I_D_ = 0.62 × AnFD^2/3^𝑣^−1/6^C√ω (1)

where I_D_—diffusion current density (mW cm^−2^); A—the geometric disk area (cm^2^); F—Faraday’s constant (C mol^−1^); D—O_2_ diffusion coefficient in the electrolyte (cm^2^ s^−1^); 𝑣—electrolyte kinematic viscosity (cm^2^ s^−1^); C—O_2_ concentration in the electrolyte (mol cm^−3^); ω—the angular frequency of rotation (rad s^−1^); and n—the average electron-transferred number contributed from the ORR.

Accordingly, the average e-transferred number can be obtained from Figure 8c, which was found to be around 3.81, which is very close to the theoretical value of four, the complete ORR. If the 2e- and 4e-routes are the only two types of the ORR, we have about 10% of the 2e-route and 90% of the 4e-route of the ORR for the CoNC-1000A900 cathode catalyst.

#### 3.7.2. Polarization Curve and Power Density Curve Measurements

**Figure 9:**
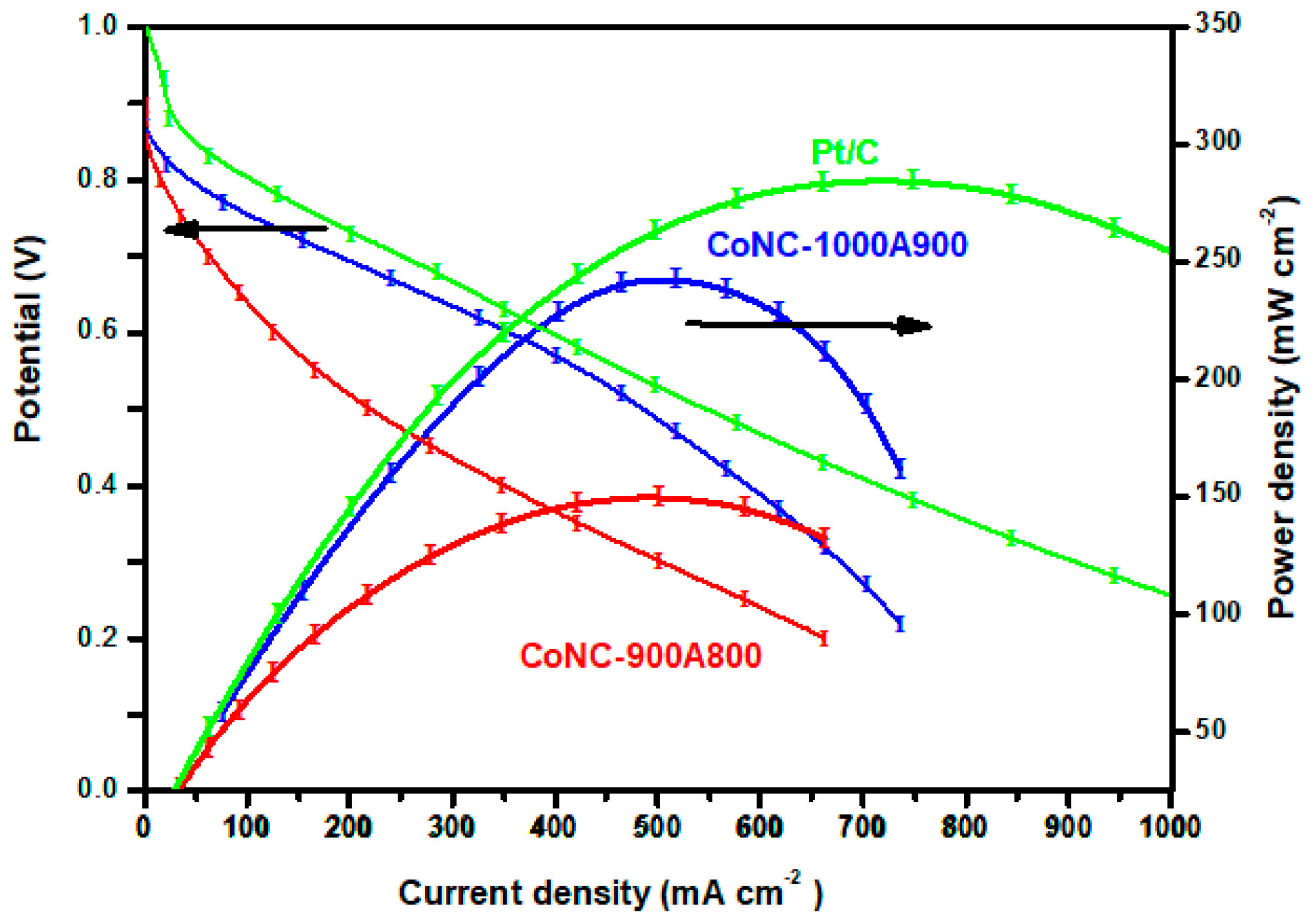
Polarization and power density curves of various Co-N-Cs.

The previous section illustrated that Co-N-Cs could be a promising cathode catalyst for the ORR. Commercial Pt/C and different Co-N-Cs were used as the cathode catalysts in an MEA, using an OH^−^ ion-conveying membrane as the solid-state electrolyte. Similar to the set-up of the metal electrodes mentioned in Ref. [33], we assembled an MEA for the single-cell station and the I–V polarization curves and power density were measured and demonstrated in Figure 9.

Most of the single cell of the AEMFC using Co-N-C as a cathode catalyst does not demonstrate a P_max_ higher than 150 mWcm^−2^ if the anode is made of Pt/C. The P_max_ of the single cell based on the CoNC-900A800 cathode catalyst can achieve up to 150 mW cm^−2^ [34,35,36,37] and can reach even higher (243 mW cm^−2^) when the CoNC-1000A900 was cathode catalyst, approaching the P_max_ of the single cell using precious Pt/C (285 mW cm^−2^), according to Figure 9. These results emphasize that a cathode catalyst with a significant ORR capability (exhibiting a significant ORR peak, high reduction current, and low resistance) is critical to obtaining an AEMFC with high power generation capacity.

#### 3.7.3. Electrochemical Stability

**Figure 10:**
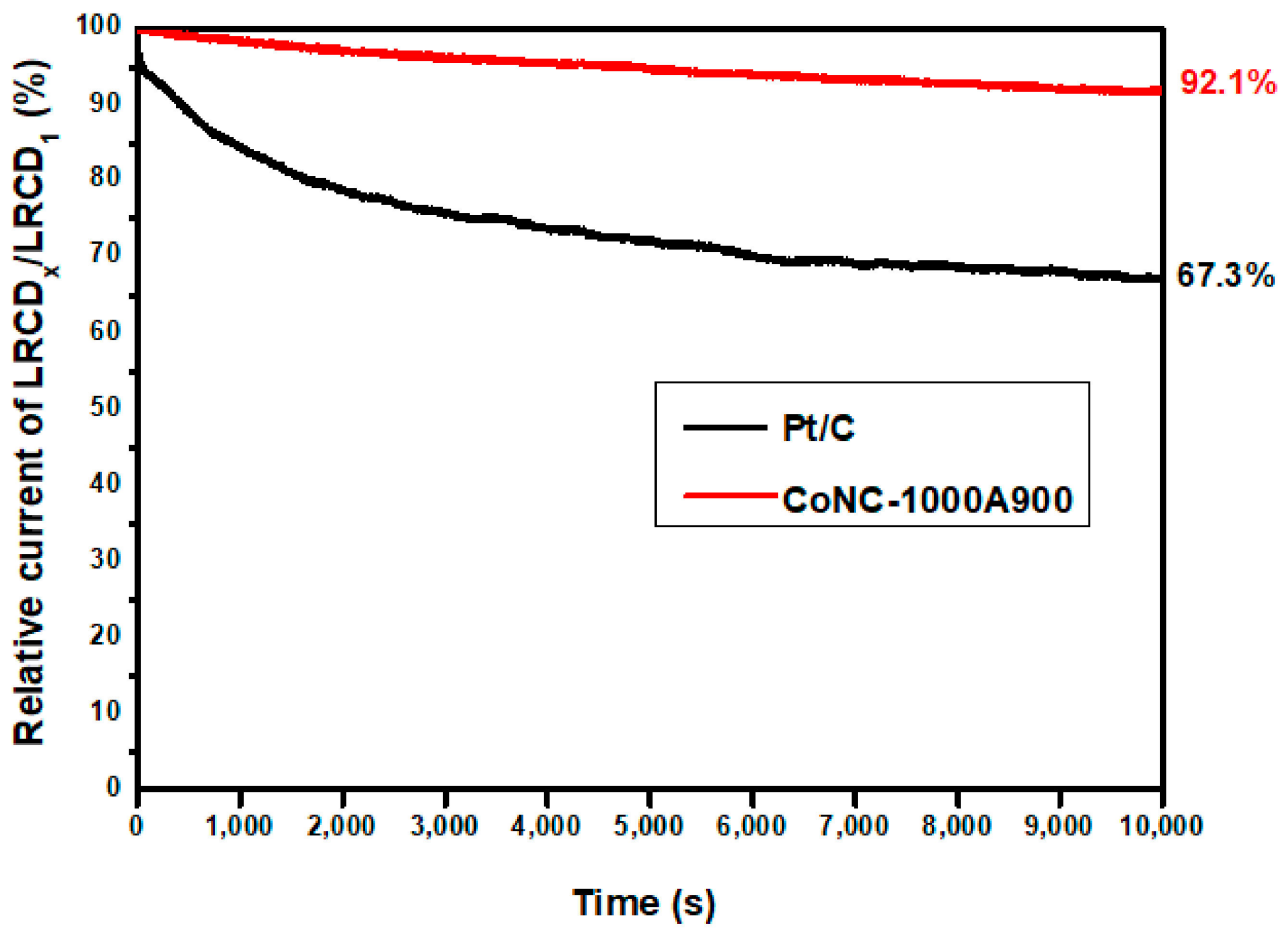
Electrochemical stability testing of Pt/C and CoNC-1000A900.

An electrochemical stability evaluation curve was constructed in 0.1 M KOH_(aq)_, recording the decadence of the LRCD during repeating redox reactions. The LRCD values obtained from the LSV curves of CoNC-1000A900 with various redox cycles are plotted vs. time recorded in seconds. The relative current is calculated from the ratio of LRCDx measured at different redox performing times compared with that of the first cycle (LRCD1) in Figure 10 to represent the electrochemical stability of the catalyst. Therefore, the number of redox cycles can be calculated by dividing the time by the cycle period. We found that we were still able to retain 92.1% of the LRCD for CoNC-1000A900 after 10,000 times of redox cycles, compared to 22.7% LRCD loss (67.3% retained) of Pt/C. The calcined Co-N-doped carbon-based (Co-N-C) catalyst demonstrated higher durability under numerous redox cycles than the commercial Pt/C, according to Figure 10.

## 4. Conclusions

We successfully prepared an N-containing polyimine with side amino groups that complex with Co(II). The Co(II)-complexed polyimines were converted into Co-N-C cathode catalysts of an AEMFC via calcination. The prepared Co-N-C catalysts demonstrated a carbon nano-belt (CNB) structure under high-temperature calcination (>800 °C) due to intra-chain crosslinking, which contributed to a BET surface area higher than 400 m^2^ g^−1^. The Co-N-C catalysts demonstrated a significant ORR peak and higher limited reduction current than that of commercial Pt/C, resulting from the lower Tafel slope and higher carbon matrix conductivity. The single cell based on the CoNC-1000A900 cathode catalyst gradually produced a P_max_ of 243 mW cm^−2^, which was slightly lower than that of Pt/C, but much higher than other AEMFCs that were also using Co-N-Cs as their cathode catalysts.

Future work will focus on removing metals from the catalyst, considering the contamination from transition metals, and preparing a cathode catalyst free of metals.

## Figures and Tables

**Table 1:** I_D_/I_G_ ratios and volume resistivity of Co-N-Cs.

CoNC-	I_D_/I_G_	ρ (Ω cm)
800A700	1.072	221.6
900A800	0.941	74.7
1000A900	0.907	29.7
1000	0.866	9.3

**Table 2:** Specific surface area and pore size distribution of Co-N-Cs prepared at different temperatures.

CoNC-	BET Surface Area (m^2^ g^−1^)	Pore Diameter (nm)
800A700	342.07	4.09
900A800	335.13	7.4
1000A900	437.80	5.3

**Table 3:** Various electrochemical properties of Co-N-C catalysts.

Catalyst	Half-Wave Potential (V) ^a^	LRCD (mA cm^−2^) ^a^	Tafel Slope (mV dec^−1^)	E_onset_ (V) ^a^
CoNC-800A700	0.62	−2.72	183.8	-
CoNC-900A800	0.72	−3.92	91.4	0.870
CoNC-1000A900	0.75	−5.80	82.6	0.897
Pt/C	0.81	−5.26	75.9	1.005

^a^: obtained from Figure 7b.

## Data Availability

Not applicable.
